# Complete Chloroplast Genome of *Crassula aquatica*: Comparative Genomic Analysis and Phylogenetic Relationships

**DOI:** 10.3390/genes15111399

**Published:** 2024-10-30

**Authors:** Kyu Tae Park, OGyeong Son

**Affiliations:** 1 Department of Life Sciences, Yeungnam University, Gyeongsan 38541, Republic of Korea; allan57@ynu.ac.kr; 2 Planning & Budget Office, Management Planning Division, Daegu National Science Museum, Yugaeup, Daegu 43023, Republic of Korea

**Keywords:** *Crassula aquatica*, Crassulaceae, chloroplast genome, *rpoC1* intron loss, codon usage, phylogeny

## Abstract

Background/Objectives: *Crassula aquatica* (L.) Schonl. is a very small annual plant growing along riverbanks. Chloroplast (cp) genomes, crucial for photosynthesis, are highly conserved and play a key role in understanding plant evolution. In this study, we conducted cp genome analysis of *C. aquatica*, aiming to elucidate its phylogenetic position and structural variations. We analyzed and described the features of the complete cp genome of *C. aquatica* and conducted comparative analysis with the cp genomes of closely related taxa. Rsults: The cp genome was 144,503 bp in length and exhibited the typical quadripartite structure, consisting of a large single-copy region (LSC; 77,993 bp), a small single-copy region (SSC; 16,784 bp), and two inverted repeats (24,863 bp). The cp genome of *C. aquatica* comprised 113 unique genes, including 79 protein-coding genes (PCGs), 30 tRNAs, and 4 rRNA genes. Comparative genomic analysis of 13 other *Crassula* species and six outgroups demonstrated highly conserved gene content and order among *Crassula* species. However, notable differences were observed, including the complete loss of the *rpoC1* intron in *C. aquatica* and several closely related species, which may serve as a synapomorphic trait supporting the monophyly of the subgenus *Disporocarpa*. We analyzed the nucleotide diversity among 14 *Crassula* cp genomes and identified five highly variable regions (*pi* > 0.08) in the IGS regions. Phylogenetic analysis based on 78 PCGs confirmed the monophyly of *Crassula* and its division into two subgenera: *Crassula* and *Disporocarpa*. Although the phylogenetic tree supported the subgeneric classification system, the sectional classification system requires reassessment. Conclusions: In this study, we conducted a comparative analysis of the cp genome of the *genus Crassula*. We inferred evolutionary trends within the *Crassula* cp genome and provided molecular evidence supporting the integration of the *genus Tillaea* into the *genus Crassula*. However, as this study does not represent all species within the *genus Tillaea,* further comprehensive phylogenetic analyses are requrired.

## 1. Introduction

The chloroplast (cp), primarily involved in photosynthesis, is considered to have originated from an endosymbiotic cyanobacterium [1]. The cp genome is typically inherited uniparentally, exists in multiple copies per cell, and evolves at a slower rate compared to nuclear and mitochondrial genomes [2]. The cp genome is a circular molecule with a quadripartite structure and two inverted repeats (IR) separated by large single-copy (LSC) and small single-copy (SSC) regions [3,4]. The majority of genes encode proteins related to photosynthesis and their expression, as well as the remaining tRNAs and rRNAs. The cp genomes of angiosperms show significant conservation in terms of gene content, order, and organization [4,5]. It is suitable for comparison across various species. Furthermore, due to maternal inheritance, the cp genome shows less genetic variation, allowing it to reflect intra-lineage variations. Additionally, as it consists of a single circular DNA, the complete sequencing of the cp genome is relatively straightforward. Thus, the cp genome is appropriate for discovering phylogenetic relationships and explaining speciation processes among species [6,7]. In particular, structural variations such as rearrangements, gene loss, or pseudonization play a role in phylogenetic studies as they reflect genetic events (such as evolutionary divergence or environmental adaptation) within specific lineage [8,9,10,11]. The cp genomes of several species of the family Crassulaceae have been reported [12,13,14]. Ding et al. [15] recently reported complete cp genome sequences of several members of the genus *Crassula*.

The family Crassulaceae consists of approximately 1500 species across 34 genera [16,17], and all species within this family are succulent, easily distinguishing them from the sister family Haloragaceae [18]. Crassulaceae is divided into three subfamilies: Crassuloideae, Kalanchoideae, and Sempervivoideae [14]. These subfamilies are further classified into seven major clades: *Crassula* (Crassuloideae), *Kalanchoe* (Kalanchoideae), and five additional clades—*Sempervium*, *Leucosedum*, *Aeonium*, *Acre*, and *Telephiu*—comprising the subfamily Sempervivoideae [14,19,20,21,22,23,24,25]. The genus *Crassula*, comprising approximately 200 recognized species, is the sole genus in the Crassuloideae clade [17], and contributes to the diversity of Crassulaceae [20,23]. The reclassification of *Crassula* recognized two subgenera: *Crassula* L. and *Disporocarpa* C.A.Mey. [17,24,25]. After revision, *Crassula* was determined to comprise 149 species in southern Africa, organized into 20 sections. Most of *Crassula* are concentrated in western and southern South Africa. Approximately 20 additional species are distributed throughout temperate regions worldwide [26,27].

Previous phylogenetic studies of Crassulaceae, including *Crassula*, have used morphological, cytological, and molecular approaches [22,23,24,25,28,29,30,31]. One of the major issues in the phylogenetic study of *Crassula* is its monophyly. While the monophyly of the subgenus *Crassula* is clearly demonstrated, the monophyly of the subgenus *Disporocarpa* remains a subject of debate [22,23]. Thus, to clarify the phylogenetic relationships of *Crassula*, it is necessary to secure more evidence through additional studies. Another major issue is the phylogenetic relationship between the genus *Crassula* and the closely related genus *Tillaea.* Tölken (1985) treated *Tillaea* as a synonym under *Crassula* [26]. However, Gilbert et al. (2000) resurrected *Tillaea* based on the high level of sequence divergence in the cp DNA data [27]. In contrast, Mort et al. [25] and Bruyns et al. [31] indicated that *Tillaea* is polyphyletically embedded across *Crassula* and suggested that *Tillaea* should be included in *Crassula*.

Our target species, *C. aquatica* (L.) Schönland, is a very small annual plant growing along riverbanks and typically grows in wet areas with sandy soil. It is distributed throughout Eurasia and North America [32]. *C. aquatica* was assessed as vulnerable (VU) in South Korea [33]. *C. aquatica* was once treated as *Tillaea aquatica* L. but was later synonymized based on previous studies [25,31]. However, in some countries the synonym *T. aquatica* is used instead of *C. aquatica* [32,33]. Thus, we sequenced, assembled, and analyzed the cp genome to better understand the phylogenetic relationships in *C. aquatica.* We anticipated that the structural variations in the *C. aquatica* cp genome would be shared with specific lineages and expected that the large sequence dataset would further clarify its relationship within the genus. This study aimed to (1) identify the genomic features of *C. aquatica*, (2) explore the cp genomic structures of *C. aquatica* and determine structural variations by comparing them with the cp genomes of 13 *Crassula* and six outgroups, and (3) elucidate the phylogenetic relationships of *C. aquatica* using 78 protein-coding genes (PCGs).

## 2. Materials and Methods

### 2.1. Sampling, DNA Extraction, and Chloroplast Genome Sequencing

Fresh tissues of *C. aquatica* were collected from Gunwigun, Republic of Korea (N 36°8′2.78″ E 128°39′47.55″) on 1 June 2023. A voucher specimen was deposited in the Herbarium of Daegu National Science Museum (DNSM) (voucher no.: DNSM22405006). Genomic DNA was extracted from the plant tissue using a DNeasy Plant Mini Kit (Qiagen Inc., Valencia, CA, USA). We outsourced sequencing to Phyzen (Seongnam, Republic of Korea), which generated 150 bp paired-end reads from a 550 bp inserts library on an Illumina NovaSeq 6000 platform (Illumina, San Diego, CA, USA).

### 2.2. Chloroplast Genome Assembly and Gene Annotation

The raw data were processed using an NGS QC Tool Kit [34], where the adaptors were trimmed and low-quality reads were filtered out using default options. Once the raw data were filtered, clean reads were de novo assembled with paired-end reads via Velvet [35]. The annotations of transfer RNA (tRNA) genes were conducted via the tRNAscan-SE search server [36]. Both PCGs and ribosomal RNAs (rRNAs) were annotated using GeSeq [37]. PCGs were considered putatively functional based on two conditions: (1) the presence of an open reading frame with a completely conserved domain, verified via the NCBI Conserved Domain Database (CDD), and (2) the absence of internal stop codons. The annotated cp genome sequence was deposited in GenBank under the accession number PQ285632. A visualization of the circular cp genome map was created using Organellar Genome DRAW (OGDRAW) [38].

### 2.3. Comparative Analyses of Chloroplast Genomes

The cp genome of *C. aquatica* was compared with 13 *Crassula* cp genomes (Table S1). To evaluate sequence similarity, cp genomes were compared using mVISTA with the LAGAN mode, producing accurate multiple alignments regardless of whether they contained inversions [39]. We used MAFFT [40] to align the cp genome sequences and analyzed the sequence divergence between *Crassula* species and *C. aquatica* using sliding window analysis to compute nucleotide variability (*pi*) in DnaSP v6.12 [41]. We applied a window size of 600 bp with a 200 bp step size. To identify cp genome rearrangements in *Crassula* species, complete cp genome alignments were performed using ProgressiveMauve v.1.1.3 [42] in Geneious Prime 2024.0.3. To identify contraction or expansion at the IR boundaries, the boundaries were compared using IRplus [43].

### 2.4. Repeat

We investigated repeat sequences, including direct, reverse, palindromic, and complementary sequences, using REPuter [44]. For repeat identification, the following parameters were used: (1) minimum repeat size of 30 bp, (2) hamming distance of 3 kb, and (3) 90% or greater sequence identity. Simple sequence repeats (SSRs) were determined using MISA-web [45] with the following minimum number of repeats: ten repeat units for mononucleotides, five units for di, four units for tri-, and three units for tetra-, penta-, and hexa-nucleotide SSRs. To investigate codon usage bias in *T. aquatica*, we used MEGA 11 [46] to calculate relative synonymous codon usage (RSCU).

### 2.5. Phylogenetic Analysis

Phylogenetic analysis was performed using 78 PCGs shared by 39 Crassulaceae cp genomes. To verify the phylogenetic relationships among the three subfamilies of Crassulaceae and to determine the precise phylogenetic position of the *genus Crassula* within the family, five Haloragaceae species and one Penthoraceae species were used as outgroups (Table S1). Gene sequences were extracted from cp genomes, aligned individually using MAFFT, and concatenated using Geneious Prime 2024.0.3. Alignments were manually examined to verify the reading frames. Maximum likelihood (ML) analysis was performed using RAxML v.8.2.4 with 1000 bootstrap replicates to evaluate the node support [47]. This analysis used the GTR+I+G model selected by jModelTest v.2.1.9 [48]. We implemented Bayesian inference using MrBayes version 3.2 [49]. To determine the best-fitting substitution model, the Akaike information criterion was implemented using jModelTest v.2.1.9. The GTR+I+G model was used in the present study. Markov chain Monte Carlo analysis was performed for 1,000,000 generations. The first 25% of the trees were discarded as burn-in, and the remaining trees were used to generate a majority-rule consensus tree. ML and Bayesian inference (BI) analyses were performed using FigTree v.1.4.3.

## 3. Results

### 3.1. Chloroplast Genome Organization

**Figure 1:**
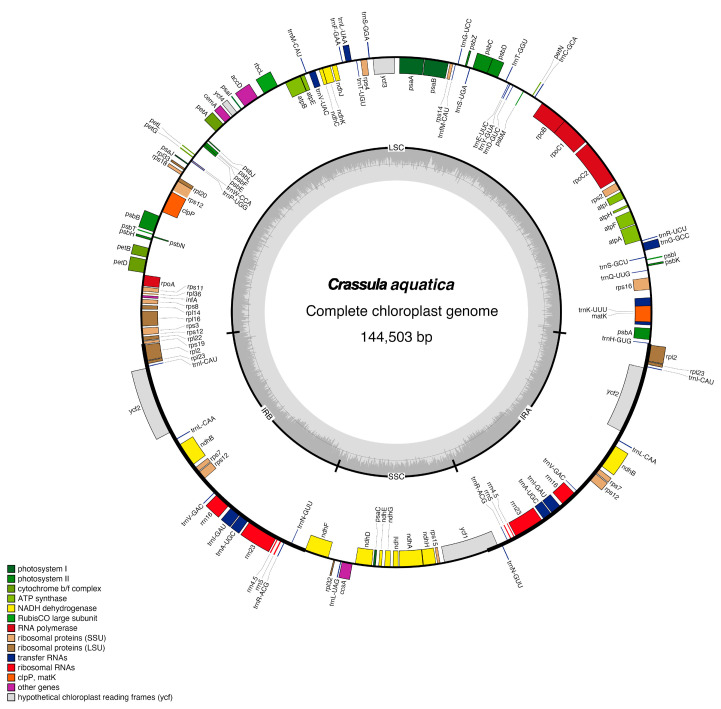
Gene map of the *C. aquatica* chloroplast genome. Genes inside the circle are transcribed clockwise, and genes outside are transcribed counterclockwise. The dark gray inner circle corresponds to the GC content, and the light gray circle corresponds to the AT content.

The length of the complete cp genome of *C. aquatica* was 144,503 bp, with a typical quadripartite structure and an LSC region (77,993 bp) separated from the SSC region (16,784 bp) by two IR regions (24,863 bp) (Figure 1, Table S2). The cp genome of *C. aquatica* comprised 113 unique genes, including 79 PCGs, 30 tRNAs, and 4 rRNAs. Among the 113 genes, 57 were related to self-replication, including 9 genes related to the large subunit of the ribosome and 12 related to the small subunit of the ribosome. A total of 43 genes were involved in photosynthesis, including 6 related to ATP synthase, 11 to NADH dehydrogenase, 6 to the cytochrome b/f complex, 5 to the PSⅠ system, 15 to the PS II system, and 1 associated with Rubisco. Additionally, nine genes were annotated as having other (*clpP*, *ccsA*, *accD*, *cemA*, and *matK*) or unknown functions (*ycf1*, *ycf2*, *ycf3*, and *ycf4*). A total of 15 genes were found to have a single intron (*atpF*, *ndhA*, *ndhB*, *petB*, *petD*, *rpl2*, *rpl16*, *rps12*, *trnA-UGC*, *trnG-GCC*, *trnI-CAU*, *trnI-GAU*, *trnK-UUU*, *trnL-UAA*, and *trnV-UAC*), while 2 genes (*clpP* and *ycf3*) contained two introns (Table S3).

### 3.2. Comparative Chloroplast Genome Structure and Polymorphism

**Figure 2:**
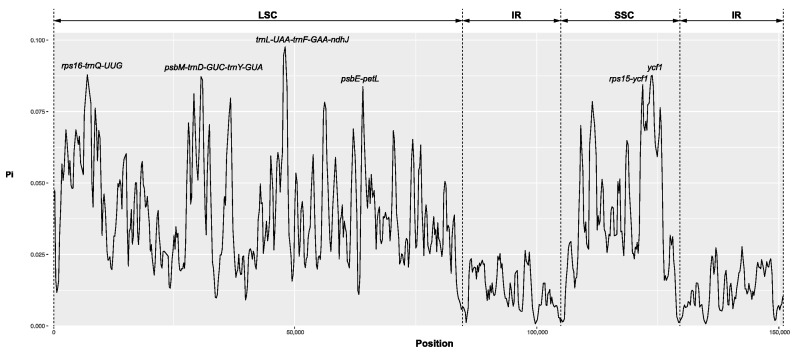
Nucleotide diversity analysis of 14 *Crassula* chloroplast genomes (window length: 600 bp; step size: 200 bp).

**Figure 3:**
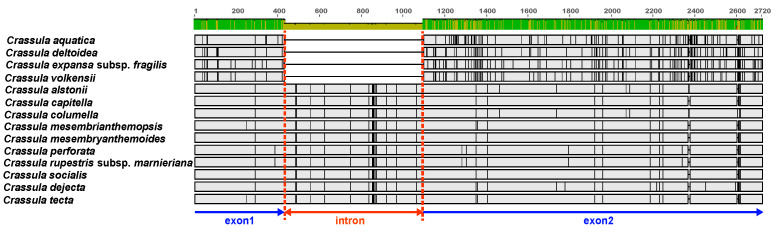
Alignment of the *rpoC1* intron loss in *Crassula*.

The entire sequence similarity of the 14 Crassula cp genomes including *C. aquatica* was evaluated using the mVISTA program. The results showed that the cp genomes of Crassula species were more conserved in the coding regions than in the non-coding regions (Figure S1). Furthermore, genomic rearrangement was not detected across the cp genome of Crassula (Figures S1 and S2). The sequence variability of the 14 Crassula cp genomes was examined by computing nucleotide polymorphisms (*pi*). The average *pi* value was estimated to be 0.03098, ranging from 0.00073 to 0.09766 (Figure 2). The sequence diversities of the IRs were more conserved (average *pi* = 0.01313) than those of the LSC (average *pi* = 0.03868) and SSC regions (average *pi* = 0.04939). In addition, the highly variable regions (*pi* > 0.08) were identified as five IGS regions (*rps16-trnQ*, *pi* = 0.08786. psbM-trnD-trnY, *pi* = 0.0.08588; trnL-trnF-ndhJ, *pi* = 0.09766; *psbE-petL*, *pi* = 0.08372; rps15-ycf1, *pi* = 0.08445) and one protein-coding region (*ycf1*, *pi* = 0.08766). We identified the IR boundaries among 14 Crassula cp genomes. Gene content and order were conserved in Crassula (Figure S3). In *C. aquatica*, the LSC/IRb boundary (J_LB_) was located on rps19, and the LSC/IRa boundary (J_LA_) was located between *Ψrps19* and trnH. The IRa/SSC and IRb/SSC boundaries (J_SA_ and J_SB_) were located on ycf1 or between the 5′ end of truncated *ycf1* and *ndhF*. The IR junction patterns were similar across all Crassula species when the IR region was compared. The length of the IR region ranged from 24,810 to 24,878 bp, and the gene content of the IR region was conserved across all Crassula species. Notably, in the *rps19* gene, despite being located in the J_LB_ in all species, in *C. aquatica*, only 33 bp were situated within the IRb region, showing a difference compared to other *Crassula* species, which included 110 bp. In the *trnH* gene, it spanned the J_LA_ in most *Crassula* species but was located in the LSC in *C. aquatica*. We found that the *rpoC1* intron was completely lost in *C. aquatica*. Among 14 Crassula cp genomes, the *rpoC1* intron had a length of 665 bp in most *Crassula* species, whereas in 4 Crassula (*C. deltodiea* Thunb., *C. expansa* subsp. fragilis (Baker) Toelken, *C. volkenssi* Engl., and *C. aquatica*), the *rpoC1* intron was completely lost (Figure 3 and Figure S1).

### 3.3. Tandem Repeat Sequence and Simple Sequence Repeat Analysis

**Figure 4:**
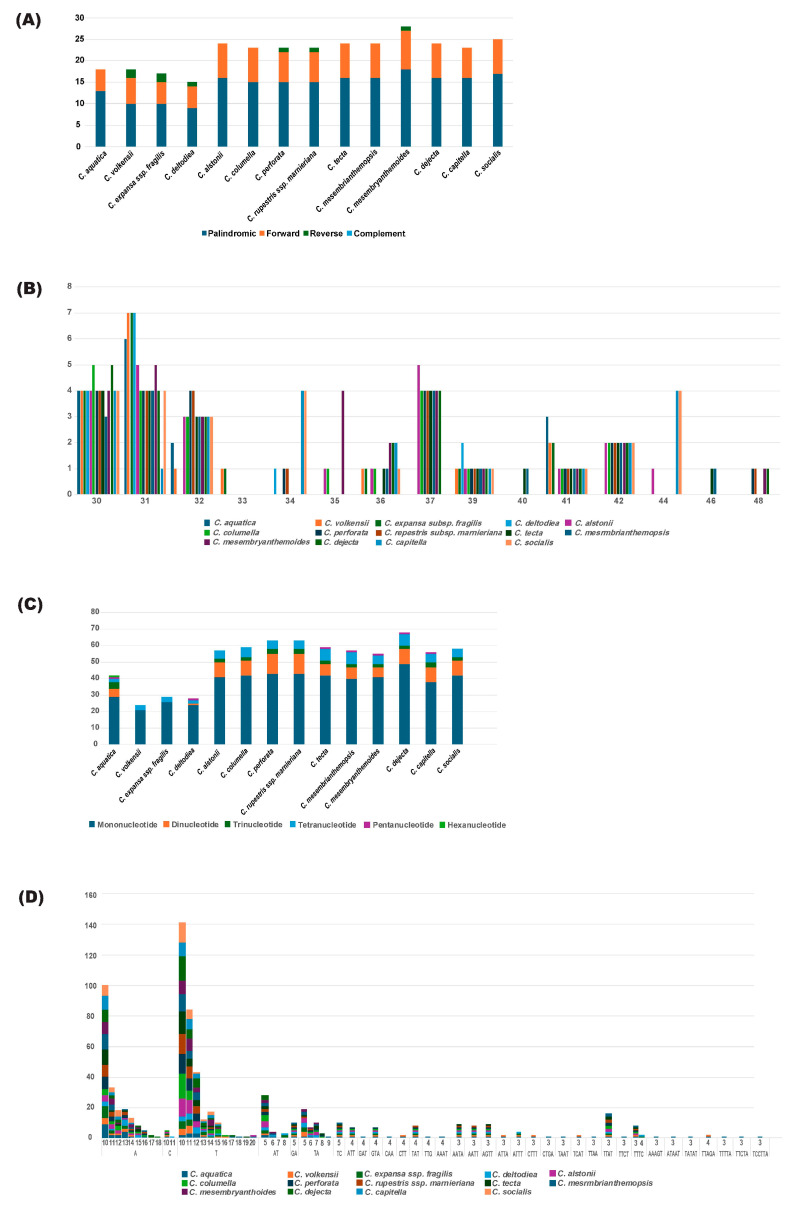
Analyses of repeated sequences in 14 *Crassula* cp genomes. (**A**) Distributions of tandem repeat types in *Crassula* cp genomes. (**B**) Frequencies of tandem repeat types in *Crassula* cp genomes. (**C**) Distributions of SSR motifs in *Crassula* cp genomes. (**D**) Frequencies of SSR motifs in *Crassula* cp genomes.

We identified 18 repeats, including 13 palindromic and 5 forward repeats in *C. aquatica*. In contrast, 15–28 repeats were identified in other *Crassula* species, including 9–18 palindromic, 5–9 forward, and 1–2 reverse repeats. No complementary repeats were detected in *Crassula* species (Figure 4A, Table S4). The length of the repeats varied from 30 to 48 bp and repeats with lengths of 30 and 31 bp were the most abundant and identified in all species, followed by those with lengths of 39, 41, and 32 bp (Figure 4B).

The total SSRs found in *C. aquatica* was 42, comprising 29 mono-, 5 di-, 4 tri-, 2 tetra-, 1 penta-, and 1 hexa-nucleotide repeats. For *Crassula*, the total number of SSRs ranged from 24 (*C. volkensii*) to 68 (*C. dejecta* Jacq.). Among these, the numbers of *Crassula* species (*C. volkensii*, *C. expansa* subsp. fragilis, and *C. deltodiea*) were lower than those of the other *Crassula* species, ranging from 24 to 29. Hexa-nucleotide repeats were detected only in *C. aquatica* (Figure 4C, Table S5). Mononucleotide repeats ranged from 21 (*C. volkensii*) to 49 (*C. dejecta*) and were most abundant in *Crassula*, with A/T repeats being the only representative (Figure 4D).

### 3.4. RSCU

**Figure 5:**
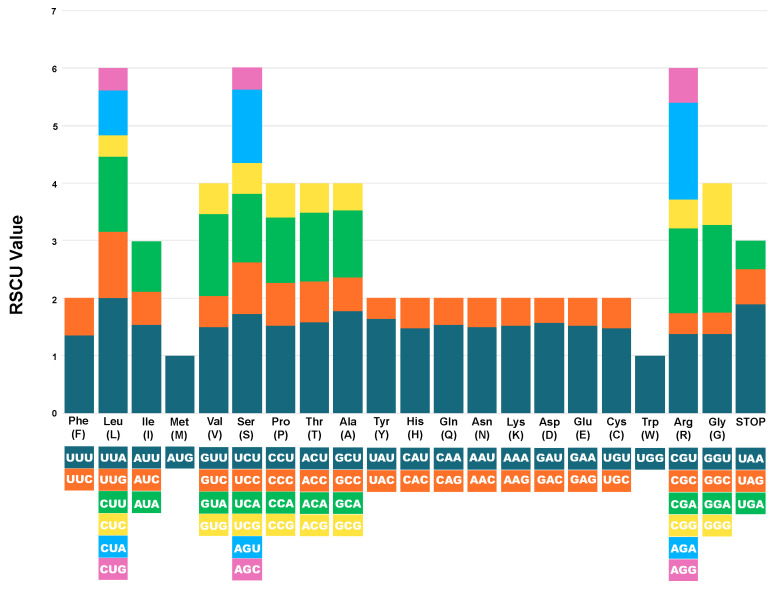
Relative synonymous codon usage (RSCU) values of 20 amino acid and stop codons in all protein-coding genes of the chloroplast genome of *C. aquatica*.

The RSCU values were calculated from the complete cp genome sequences of *C. aquatica* using all PCGs. In total, 22,408 codons (Table S4) were observed. The most abundant amino acid was leucine (Leu; 10.9%), while the least abundant was cysteine (Cys; 1.12%). The most commonly used codon was AUU (959; encodes isoleucine [Ile]), and the least used codon was UGC (65; encodes cysteine [Cys]). The RSCU frequency analysis indicated a codon usage bias. Overall, 29 amino acids had an RSCU value greater than 1, while methionine (AUG) and tryptophan (UGG) showed no codon usage bias, with an RSCU value of 1.00. The highest RSCU value was recorded for UUA (2.01; encoding Leu), and the lowest value (0.35) was recorded for UAC (encoding tyrosine [Tyr]) (Figure 5). Similar to other *Crassula* species, *C. aquatica* showed a significant preference for A/U-ending codons over G/C-ending codons in the cp genome (Figure S4, Table S4).

### 3.5. Phylogenetic Analysis

**Figure 6:**
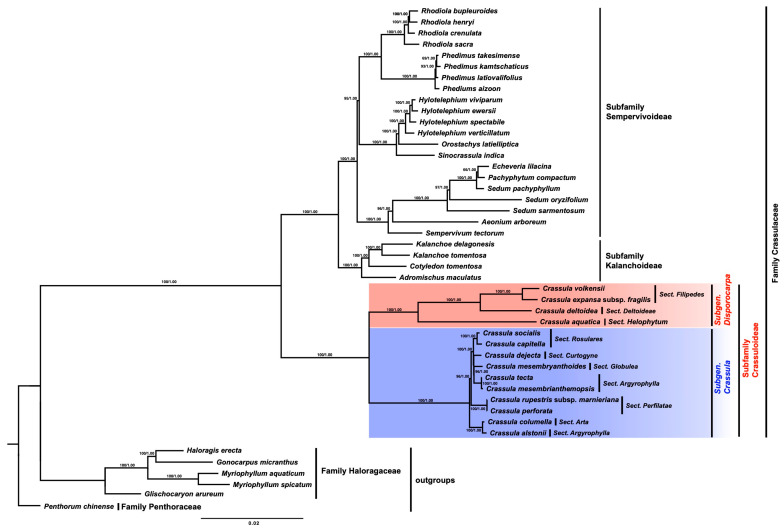
The phylogenetic tree reconstruction of 39 Crassulaceae taxa maximum likelihood based on the concatenated sequence of 78 PCGs. Numbers above the branches indicate bootstrap values and posterior probabilities.

Phylogenetic analysis was performed using 39 Crassulaceae cp genomes, including *C. aquatica* and six outgroups, based on a 65,548 bp nucleotide dataset comprising 78 PCGs (Figure 6). The topologies of the ML and BI trees were highly identical, with strong support values. All 39 Crassulaceae species were divided into three subclades, aligning with the three subfamilies (Crassuloideae, Kalanchoideae, and Sempervivoideae). Within Crassuloideae, *Crassula* formed a monophyletic group comprising two subgenera (subgenus *Crassula* and subgenus *Disporocarpa*). *C. aquatica* was clustered within the subgenus *Disporocarpa* clade with a strong support value (BS/PP = 100/1.00). Our phylogenetic tree results mostly reflected the phylogenetic relationships within the genus *Crassula*; however, *C. alstonii* Marloth (*sect*. *Argyrophylla*) did not cluster with *C. tecta* Thunb. or *C. mesembrianthemopsis* Dinter from *sect*. *Argyrophylla*. Instead, they formed a basal clade of the subgenus *Crassula* with *C. columella* Marloth and Schonland (*sect. Arta*) with a high support value (BS/PP = 100/1.00).

## 4. Discussion

When compared to the 14 *Crassula* cp genomes, the results indicated that the gene content and order of the cp genome were conserved. They showed slight variations in cp genome size and GC content. No rearrangements were observed.

The *rps19* gene was located in J_LB_ in all Crassula cp genomes. In *C. aquatica*, 33 bp of the 5′ end of the *rps19* was located in the IRb region, whereas in other species, the 5′ end of the *rps19* gene was located at 110 bp within the IRb region. Additionally, previous studies on the Crassulaceae family have consistently reported a 110 bp location [12,13,14,15]. In contrast, in the cp genome of Haloragaceae used as an outgroup, the 5′ end of the rps19 gene was located 2–3 bp within the IRb region, whereas in Penthoraceae, the entire rps19 gene was located only in the LSC region (Figure S3). This suggests an evolutionary trend of IR expansion in Crassulaceae cp genomes. IR expansion and contraction can occur via several mechanisms [50,51]. Short IR expansion can occur due to gene conversion, whereas large IR expansion can result from double-stranded DNA breaks [50]. Thus, to more accurately identify the trends in IR expansion and contraction, it is necessary to conduct further studies on *Crassula*, Crassulaceae, and related taxa.

In this study, we identified that the *rpoC1* intron was completely lost in *C. aquatica* and its related species. The loss of the *rpoC1* intron has been observed in various lineages and, in some cases, provides important phylogenetic information [52,53,54,55,56]. The *rpoC1* gene codes for a subunit of plastid-encoded RNA polymerase, an enzyme that is crucial for cp gene expression [57]. As a result of the degeneration of RNA structures and intron-encoded proteins (IEP), cp introns lose their self-splicing ability [57]. The loss of introns is considered to be a result of evolutionary adaptation, potentially occurring in scenarios where a simplified genome structure or more efficient gene expression is required [52]. Moreover, among the 14 *Crassula* cp genomes, the *rpoC1* intron was completely lost in all species of the *subgenus Disporocarpa*, including *C. aquatica*. This loss of the *rpoC1* intron is considered a synapomorphic characteristic of the *subgenus Disporocarp* and provides evidence supporting its monophyly.

Nucleotide diversity analysis of the 14 *Crassula* cp genomes revealed six highly variable regions (HVR, *pi* > 0.08), with five found in the IGS region and one in the protein-coding region. A previous study reported a total of 11 HVRs within *Crassula*, and the observations were similar, with both results indicating the highest variability in the trnL-UAA-trnF-GAA-ndhJ region [11]. These HVR with high *pi* values can potentially provide DNA barcodes for species identification within the *Crassula*.

The codon usage pattern is an important genetic trait of an organism and is related to mutations, selection, and other phenomena of molecular evolution [58,59,60,61,62]. The cp genome of *C. aquatica* was analyzed and compared with those of other *Crassula* species to investigate the patterns of codon usage. In 14 *Crassula* cp genomes, leucine showed the highest frequency among all amino acids, whereas cysteine displayed the lowest frequency, excluding the top codons. Additionally, RSCU analysis revealed that codons ending in A/U had RSCU values greater than 1, whereas those ending in C/G had RSCU values less than 1. *C. aquatica* exhibits a preference for A/U-ending codons, which is consistent with the overall AT-rich content commonly observed in angiosperm cp genomes [58,59,60,61,62]. This pattern was similar to that of other *Crassula* species, which also demonstrated a significant preference for A/U at the third codon position.

The distribution and amount of repetitive sequences in the cp or nuclear genome are likely to contain phylogenetic signals [19,63,64,65,66,67,68,69]. In this study, the number of tandem repeats in the cp genome of the *Crassula* ranged from 15 (*C. deltodiea*) to 28 (*C. mesembryanthemoides* Haw.). Species in the *subgenus Disporocarpa* (e.g., *C. aquatica*, *C. deltoidiea*, *C. volkensii*, and *C. expansa* subsp. *fragilis*) exhibited 15–18 repeats, whereas species in *subgenus Crassula* showed 23–28 repeats (Table S4). Further studies involving additional *Crassula* species are needed to clarify the relationships between the number of repeats and phylogenetic patterns. SSRs are important codominant DNA molecular markers with the benefit of high abundance, random distribution throughout the genome, and substantial polymorphism data [63,64,65,66,67,68,69]. Therefore, they can offer significant insights into various areas such as populations genetics, phylogeography, and species identification [69]. A total of 718 SSRs were identified in 14 *Crassula* cp genomes, with *C. dejecta* containing the highest number. In each genome, A/T was the predominant motif among mononucleotide SSRs with the highest frequency. In contrast, hexa-nucleotide SSR motifs were identified only in *C. aquatica* within *Crassula* (Table S5). The microsatellites discovered in this study can be developed as markers for *C. aquatica*, contributing to future research on species identification and evolutionary studies within this genus.

In this study, the phylogenetic analysis results revealed that the phylogenetic tree was split into three subfamilies and six clades, aligning with findings from previous Crassulaceae phylogenetic studies [12,13,14,21]. The genus *Crassula* was validated as a monophyletic group with strong support values (BS = 100/PP = 1.00). In addition, they were grouped into two subgenera, supporting the subgeneric classification system proposed by Tölken [24]. However, the following exceptions were identified at the sectional level: *C. alstonii* (sect. Argyrophylla) was not grouped with *C. tecta* or *C. mesembrianthemopsis* in the same section; instead, it was closely related to *C. mesembryanthemooides* (sect. Globulea). This suggests potential issues with the sectional classification proposed by Tölken [26]. Furthermore, in a study by Bruyns et al. [26], only 5 of 20 sections (*sect. Petrogeton—subgenus Disporocara; sect. Acutifolia*, *sect. Subulares*, *sect. Kalosanthes*, and *sect. Columnares—subgenus Crassula*) were confirmed to be monophyletic. These results indicate that although the subgeneric classification is generally valid, the sections exhibit a paraphyletic pattern, suggesting the need for a more detailed reassessment [26,31]. According to the current classification, *C. aquatica* belongs to the *subgenus Disporocarpa*, *section Helophytum*. Our results also showed that *C. aquatica* was grouped within the *subgenus Disporocarpa* and was closely related to *C. deltoidei* (*sect. Deltoideae*) among the 13 *Crassula* species analyzed. These results are consistent with the pattern observed in *Crassula* chloroplast genomes (*rpoC1* intron loss), providing strong evidence that *C. aquatica* belongs to the *subgenus Disporocarpa*. *Tillaea* was initially recognized as a specific species within the *genus Crassula* but was treated as a synonym by Tölken [26]. Using AFLP data, Ham and t’Hart [30] found that a single sampled species of *Tillaea* was closely related to two *Crassula* species. Based on these results, Gilbert et al. [27] classified certain *Crassula* species as *Tillaea*, despite a very limited sample size. However, Mort et al. [25] and Bruyns et al. [31] revealed that *Tillaea* species are not monophyletic but are instead embedded in different clades. The controversial taxonomic position and polyphyletic pattern of *Tillaea* in previous studies are likely due to a lack of understanding of these species (resulting from limited or insufficient data) and have led to an inaccurate reflection of their relationships. By obtaining more data, such as additional sequences data or taxa, these issues can be resolved. Although only one taxon, *C. aquatica*, previously classified under *Tillaea*, was included in this study, our results based on a large nucleotide sequence data set and a comparison of cp genome structure indicated its inclusion within the genus *Crassula*. Moreover, the results demonstrated an evolutionary trend, with structural variations shared exclusively within the *subgenus Disporocarpa*. Therefore, it is not reasonable to treat *Tillaea* as an independent genus, and it should be integrated into the *genus Crassula*.

## 5. Conclusions

In this study, we sequenced and analyzed the cp genome of *C. aquatica*. The comparative analyses revealed characteristics of the *Crassula* cp genome. Even though the cp genome size, genome structure, and gene contents of *C. aquatica* were similar to other *Crassula* cp genomes, the IR expansion, *rpoC1* intron loss, and distribution of repeats demonstrated the evolutionary history of *Crassula*. Our phylogenetic analyses supported *Crassula* being monophyletic. However, the phylogenetic analysis supported the subgeneric classification system, but the sectional classification system requires reassessment.

Meanwhile, *C. aquatica*, which was synonymized from the *genus Tillaea*, shared structural variations in the cp genome with species of the *subgenus Disporocarpa*, and phylogenetic analysis showed the same pattern. This provides evidence supporting the inclusion of *Tillaea* within the *genus Crassula*. However, since only *C. aquatica* was included in this study among the taxa previously classified under *Tillaea*, it cannot represent all species of *Tillaea*. Additionally, the species of the genus *Tillaea* have now been merged into the *genus Crassula*, distributed across *subgenus Crassula* and *subgenus Disporocarpa* [17]. Therefore, to clarify the phylogenetic relationship between *Tillaea* and *Crassula*, further analysis including more taxa is necessary.

## Data Availability

The sequence data generated in this study are available in GenBank of the National Center for Biotechnology Information (NCBI) under the access number PQ285632.

## Supplementary Materials

The following supporting information can be downloaded at: https://www.mdpi.com/article/10.3390/genes15111399/s1, Figure S1: Structure comparisons of 14 Crassula chloroplast genomes using the mVISTA; Figure S2: Whole-genome alignment of 14 Crassula chloroplast genomes; Figure S3: Comparison of the boundaries of LSC, SSC, and IR regions among 14 Crassula chloroplast genomes; Figure S4: The heatmap of overall RSCU values among 14 Crassula species based on 53 chloroplast genes (length > 300 bp); Table S1: List taxa included in analysis; Table S2: Characteristics of *C. aquatica* chloroplast genome; Table S3: List of gene contents in *C. aquatica*; Table S4: Number of tandem repeat types in Crassula; Table S5: Number of SSR types in Crassula; Table S6: The RSCU values of codons among Crassula species.
