# Supplementary material for: Complete Chloroplast Genome of Crassula aquatica: Comparative Genomic Analysis and Phylogenetic Relationships
Source: Genes (Basel). 2024 Oct 30;15(11):1399. doi: 10.3390/genes15111399 (PMC11594095; doi:10.3390/genes15111399)
Supplement: Supplementary file 1 [file genes-15-01399-s001.zip › data/Supplementary/Supplementary_file.docx]

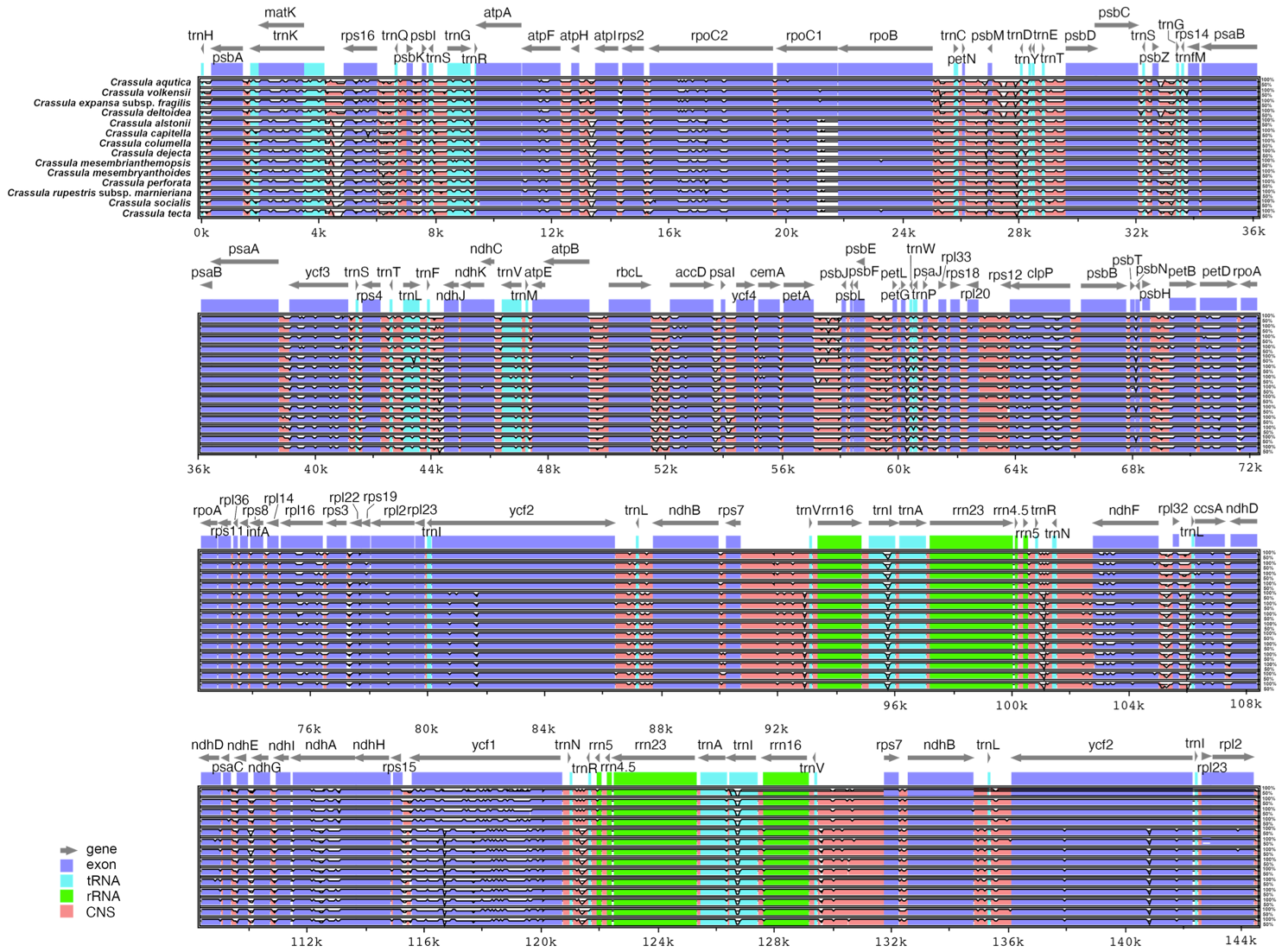


**Figure S1**. Structure comparisons of 14 *Crassula* chloroplast genomes using the mVISTA program. The vertical scale indicates the percent identity, ranging from 50% to 100%. Arrows indicate the direction of transcription of annotated genes in the reference genome.


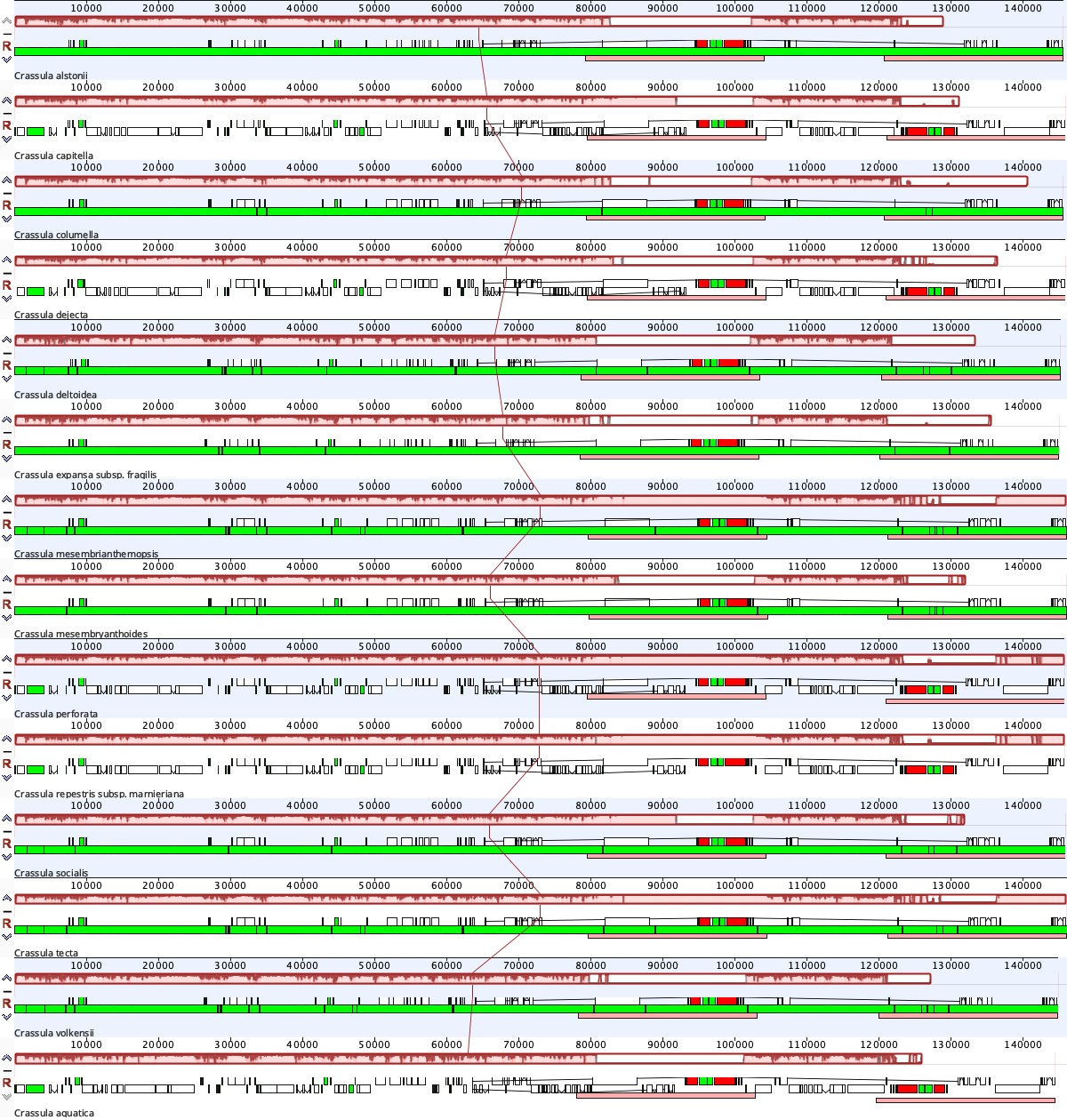


**Figure S2**. Whole-genome alignment of 14 *Crassula* chloroplast genomes. The height of the colored region with a block reflects the average sequence identity.

**Figure S3.** Comparison of the boundaries of LSC, SSC, and IR regions among 14 *Crassula* chloroplast genomes.

**Figure S4.** The heatmap of overall RSCU values among 14 *Crassula* species based on 53 chloroplast genes (length > 300bp). The x-axis: the clusters of species, y-axis: the clusters of different codons.

**Table S1.** List taxa including in analysis

| Species | Assession number |
| --- | --- |
| *Adromischus maculatus* | OM678464 |
| *Aeonium arboreum* | MW206792 |
| *Cotyledon tomentosa* | MW848817 |
| *Crassula aquatica* | PQ285632 |
| *Crassula alstonii* | OP729482 |
| *Crassula capitella* | OQ076650 |
| *Crassula columella* | OP729483 |
| *Crassula dejecta* | OP729484 |
| *Crassula deltoidea* | OP882298 |
| *Crassula expansa subsp. fragilis* | OP882299 |
| *Crassula mesembrianthemopsis* | OP882297 |
| *Crassula mesembryanthoides* | OP729485 |
| *Crassula perforata* | MW206794 |
| *Crassula rupestris* subsp*. marnieriana* | OM935751 |
| *Crassula socialis* | OP729486 |
| *Crassula tecta* | OP729487 |
| *Crassula volkensii* | OP882300 |
| *Echeveria lilachina* | MZ643065 |
| *Hylotelephium ewersii* | MN794014 |
| *Hylotelephium spectabile* | NC_072128 |
| *Hylotelephium viviparum* | OK094424 |
| *Kalanchoe delagonesis* | MW237833 |
| *Kalanchoe tomentosa* | MN794319 |
| *Orostachys latielliptica* | ON979328 |
| *Pachyphytum compactum* | MW206798 |
| *Phedimus kamtschaticus* | MG680403 |
| *Phedimus latiovalifolius* | OP344952 |
| *Phediums aizoon* | MN794321 |
| *Rhodiola bupleuroides* | MT460447 |
| *Rhodiola crenulata* | MK281587 |
| *Rhodiola henryi* | MT460449 |
| *Rhodiola sacra* | OP312066 |
| *Sedum pachyphyllum* | OP310967 |
| *Sedum sarmentosum* | JX427551 |
| *Sedum takesimense* | KF954541 |
| *Sedum taquetii* | OP537245 |
| *Sempervivum tectorum* | NC_053954 |
| *Sinocrassula indica* | MN794334 |
| *Myriophyllum spicatum* | MH191392 |
| *Myriophyllum aquaticum* | OP231037 |
| *Gonocarpus micranthus* | MW971559 |
| *Glischocaryon arureum* | MW971555 |
| *Haloragis erecta* | MW971558 |
| *Penthorum chinense* | JX436155 |

**Table S2**. Characteristics of *C. aquatica* chloroplast genome

| Characteristics | Number |
| --- | --- |
| Total length (bp) | 144,443 |
| Length of Large single-copy (LSC)region (bp) | 77,993 |
| Length of Small single-copy (SSC)region (bp) | 16,784 |
| Length of inverted repeat (IR)(bp) | 24.863 |
| Total GC contents (%) | 38.3 |
| No. of protein coding genes | 79 |
| No. of tRNA genes | 30 |
| No. of rRNA genes | 4 |
| No. of unique genes | 113 |

**Table S3**. List of gene contents in *C. aquatica*

| **Category** | **Groups of Genes** | **Names of Genes** | |
| --- | --- | --- | --- |
| **Self-replication** | **Ribosomal RNA** | rrn4.5†, rrn5†, rrn16†, rrn23† | |
|  | **Transfer RNA** | *trnA-UGC^†,‡^*, *trnC-GCA, trnD-GUC, trnE-UUC, trnF-GAA, trnfM-CAU, trnG-GCC^‡^, trnG-UCC, trnH-GUG, trnI-CAU^†^, trnI-GAU^†,‡^, trnK-UUU^‡^, trnL-CAA^†^, trnL-UAA^‡^, trnL-UAG, trnM-CAU, trnN-GUU†, trnP-UGG, trnQ-UUG, trnR-ACG†, trnR-UCU, trnS-GCU, trnS-GGA, trnS-UGA, trnT-GGU, trnT-UGU, trnV-GAC^†^, trnV-UAC^‡^, trnW-CCA, trnY-GUA* | |
|  | **Small subunit of ribosome** | *rps2, rps3, rps4, rps7^†^, rps8, rps11, rps12^†,‡^, rps14, rps15, rps16, rps18, rps19* | |
|  | **Large subunit of ribosme** | *rpl2^†,‡^, rpl14^†^, rpl16^†,‡^, rpl20, rpl22^†^, rpl23^†^, rpl33, rpl36* | |
|  | **RNA polymerase subunit** | *rpoA, rpoB, rpoC1, rpoC2* | |
| **Photosynthesis**  **Others** | **ATP synthase** | *atpA, atpB, atpE, atpF^‡^, atpH, atpI* | |
|  | **NADH dehydrogenase** | *ndhA^‡^, ndhB^†,‡^, ndhC, ndhD, ndhE, ndhF, ndhG, ndhH, ndhI, ndhJ, ndhK* | |
|  | **Cytochrome b/f complex** | *petA, petB^‡^, petD^‡^, petG, petL, petN* | |
|  | **PhotosystemⅠ** | *psaA, psaB, psaC, psaI, psaJ* | |
|  | **PhotosystemⅡ** | *psbA, psbB, psbC, psbD, psbE, psbF, psbH, psbI, psbJ, psbK, psbL, psbM, psbN, psbT, psbZ* | |
|  | **Rubisco large subunit** | *rbcL* | |
| **Others** | **Translation initiation** | *infA* | |
|  | **Proteolysis** | *clpP^‡^* | |
|  | **Cytochrome *c* biogenesis protein** | *ccsA* | |
|  | **Acetyl-CoA carboxylase** | *accD* | |
|  | **Chloroplast envelope membrane protein** | *cemA* | |
|  | **maturase** | *matK* | |
|  | **Hypothetical reading frame** | *ycf1, ycf2^†^, ycf3^‡^, ycf4* | |
| †, duplicated gene in IR; ‡, gene including one or two introns | | | |

**Supplemental Table S4**. Number of tandem repeat types in *Crassula*

| Species | Repeat type | | | |
| --- | --- | --- | --- | --- |
|  | Palindromic | Forward | Reveres | Complement |
| *C. aquatica* | 13 | 5 | 0 | 0 |
| *C. volkensii* | 10 | 6 | 2 | 0 |
| *C. expansa subsp. fragilis* | 10 | 5 | 2 | 0 |
| *C. deltodiea* | 9 | 5 | 1 | 0 |
| *C. alstonii* | 16 | 8 | 0 | 0 |
| *C. columella* | 15 | 8 | 0 | 0 |
| *C. perforata* | 15 | 7 | 1 | 0 |
| *C. repestris subsp. marnieriana* | 15 | 7 | 1 | 0 |
| *C. tecta* | 16 | 8 | 0 | 0 |
| *C. mesrmbrianthemopsis* | 16 | 8 | 0 | 0 |
| *C. mesembryanthoides* | 18 | 9 | 1 | 0 |
| *C. dejecta* | 16 | 8 | 0 | 0 |
| *C. capitella* | 16 | 7 | 0 | 0 |
| *C. socialis* | 17 | 8 | 0 | 0 |

**Supplemental Table S5**. Number of SSR types in *Crassula*

| Species | Repeat type | | | | | |
| --- | --- | --- | --- | --- | --- | --- |
|  | Mono- | Di- | Tri- | Tetra- | Penta- | Hexa- |
| *C. aquatica* | 29 | 5 | 4 | 2 | 1 | 1 |
| *C. volkensii* | 21 | 0 | 0 | 3 | 0 | 0 |
| *C. expansa* subsp*. fragilis* | 26 | 0 | 0 | 3 | 0 | 0 |
| *C. deltodiea* | 24 | 1 | 0 | 2 | 1 | 0 |
| *C. alstonii* | 41 | 9 | 2 | 5 | 0 | 0 |
| *C. columella* | 42 | 9 | 2 | 6 | 0 | 0 |
| *C. perforate* | 43 | 12 | 3 | 5 | 0 | 0 |
| *C. rupestris* subsp*. marnieriana* | 43 | 12 | 3 | 5 | 0 | 0 |
| *C. tecta* | 42 | 7 | 2 | 7 | 1 | 0 |
| *C. mesrmbrianthemopsis* | 40 | 7 | 2 | 7 | 1 | 0 |
| *C. mesembryanthoides* | 41 | 6 | 2 | 5 | 1 | 0 |
| *C. dejecta* | 49 | 9 | 2 | 7 | 1 | 0 |
| *C. capitella* | 38 | 9 | 3 | 5 | 1 | 0 |
| *C. socialis* | 42 | 9 | 2 | 5 | 0 | 0 |
