# Supplementary figures and images for: Complete Chloroplast Genome of Crassula aquatica: Comparative Genomic Analysis and Phylogenetic Relationships
Source: Genes (Basel). 2024 Oct 30;15(11):1399. doi: 10.3390/genes15111399 (PMC11594095; doi:10.3390/genes15111399)

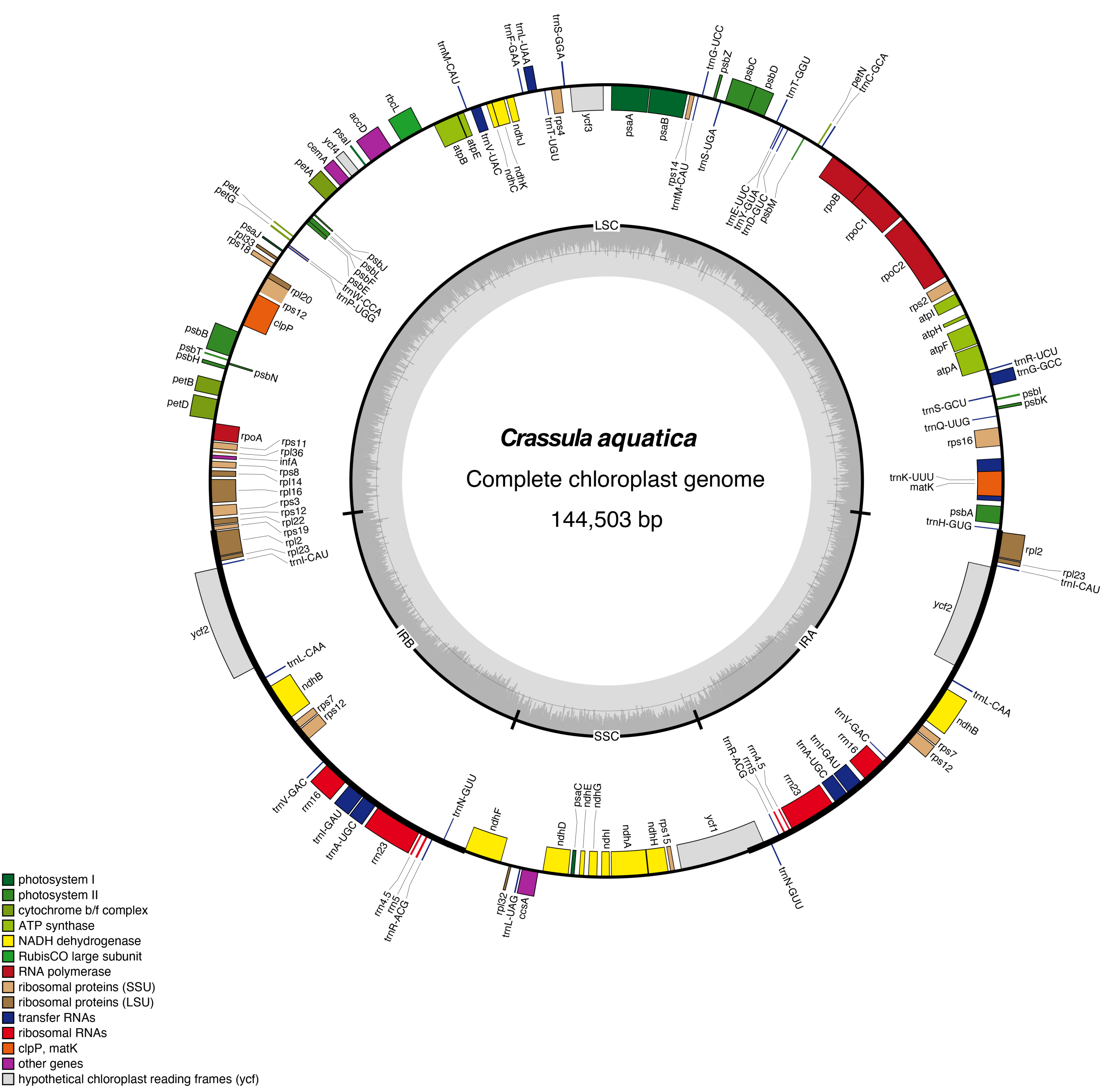

Supplement: Supplementary file 1 [file genes-15-01399-s001.zip › data/figure/Figure_1.pdf]

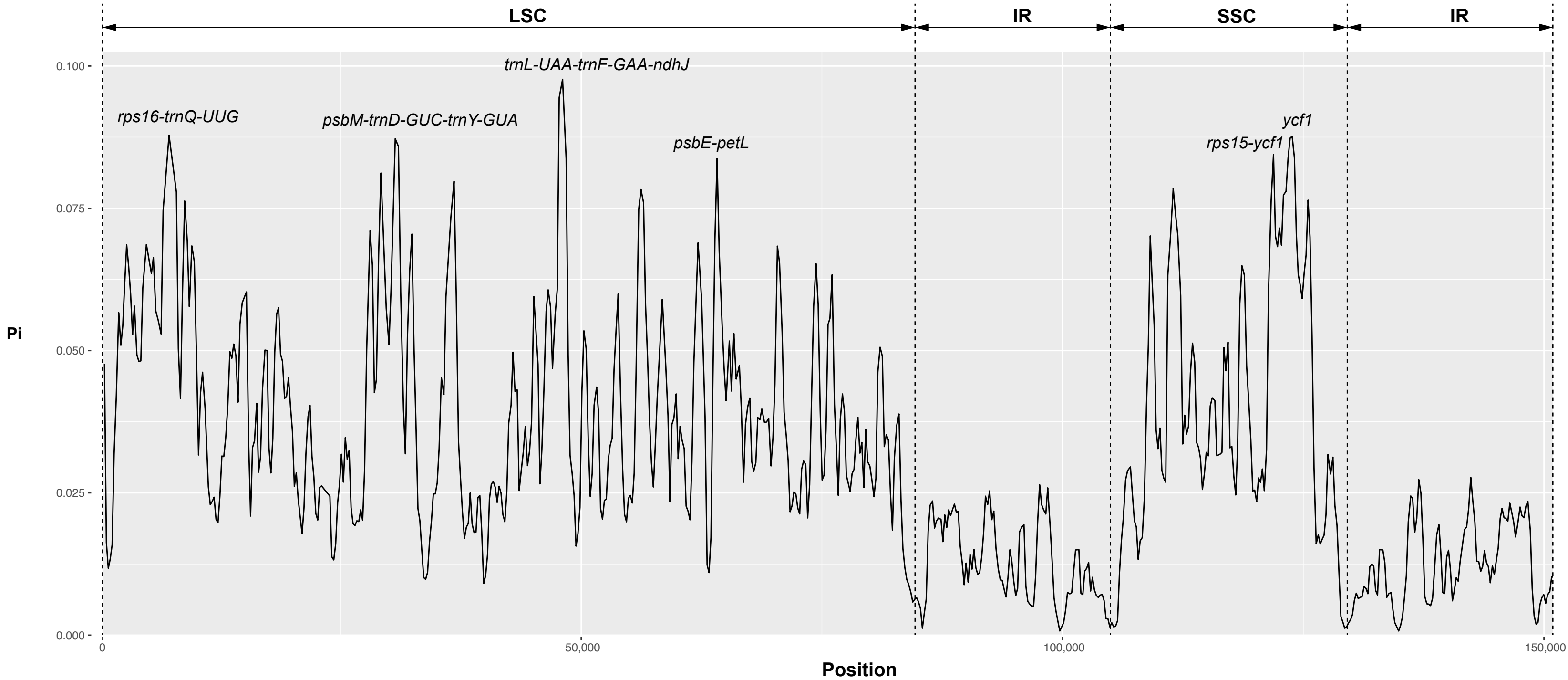

Supplement: Supplementary file 1 [file genes-15-01399-s001.zip › data/figure/Figure_2.pdf]

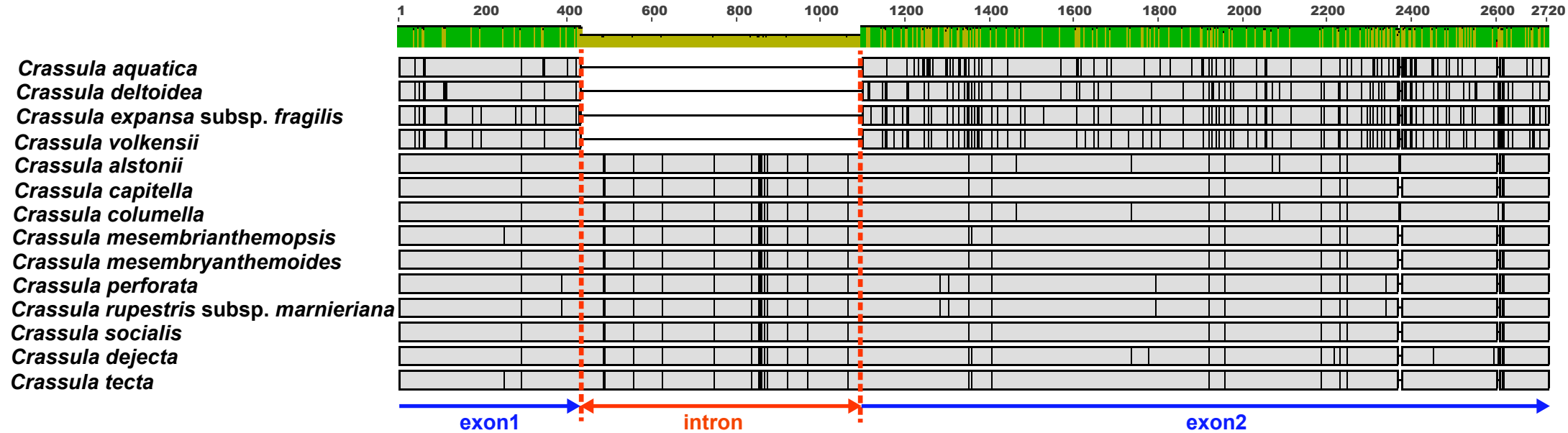

Supplement: Supplementary file 1 [file genes-15-01399-s001.zip › data/figure/Figure_3.pdf]

**(A)**

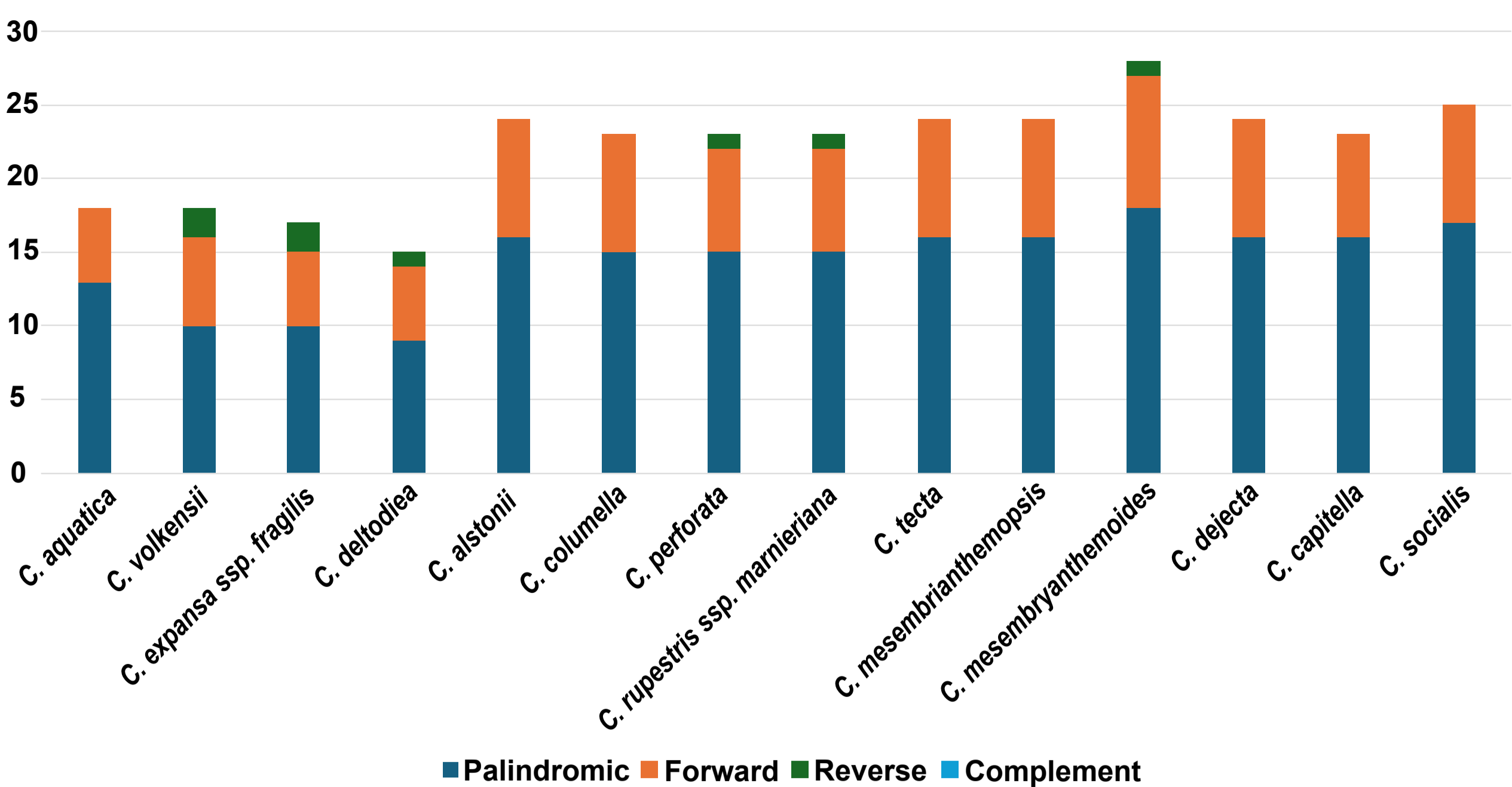

(B)

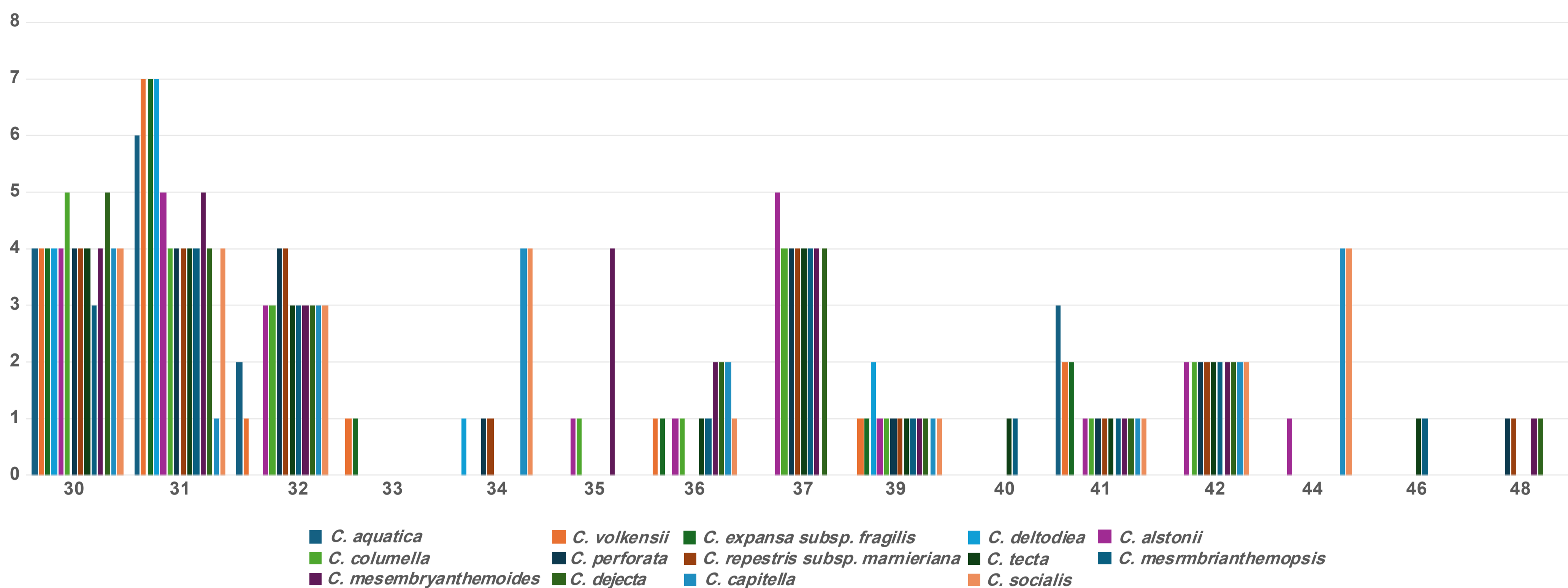

(C)

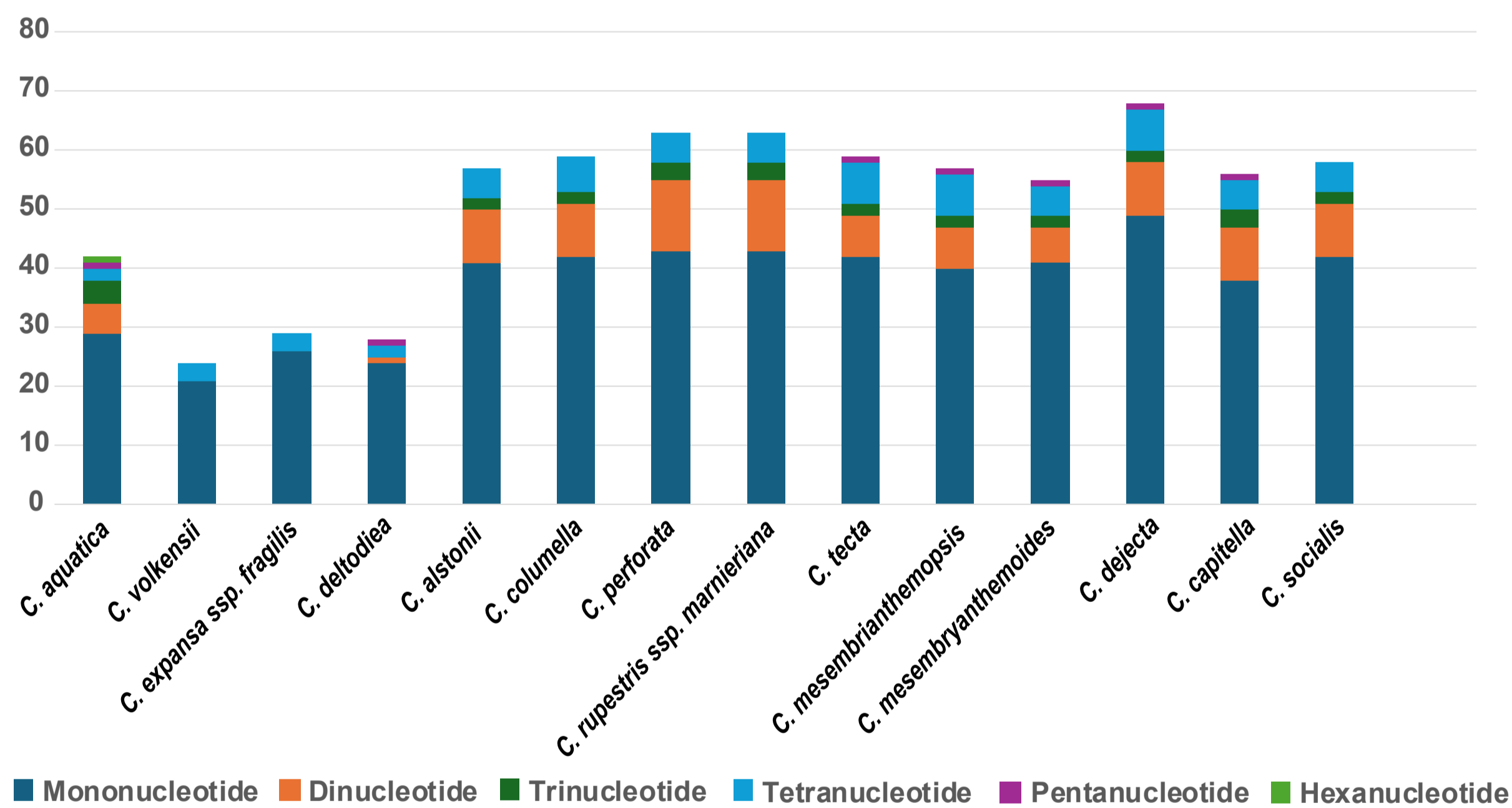

**(D)**

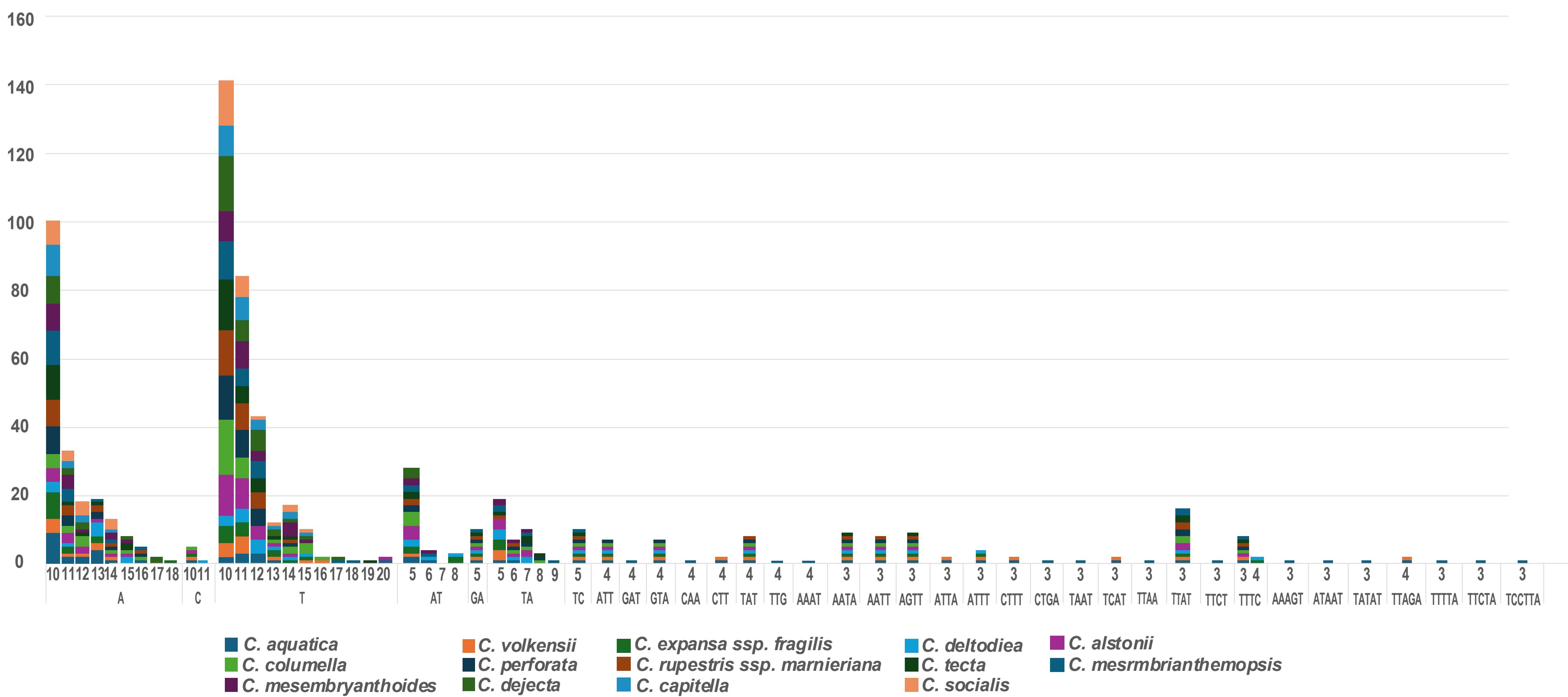

Supplement: Supplementary file 1 [file genes-15-01399-s001.zip › data/figure/Figure_4.pdf]

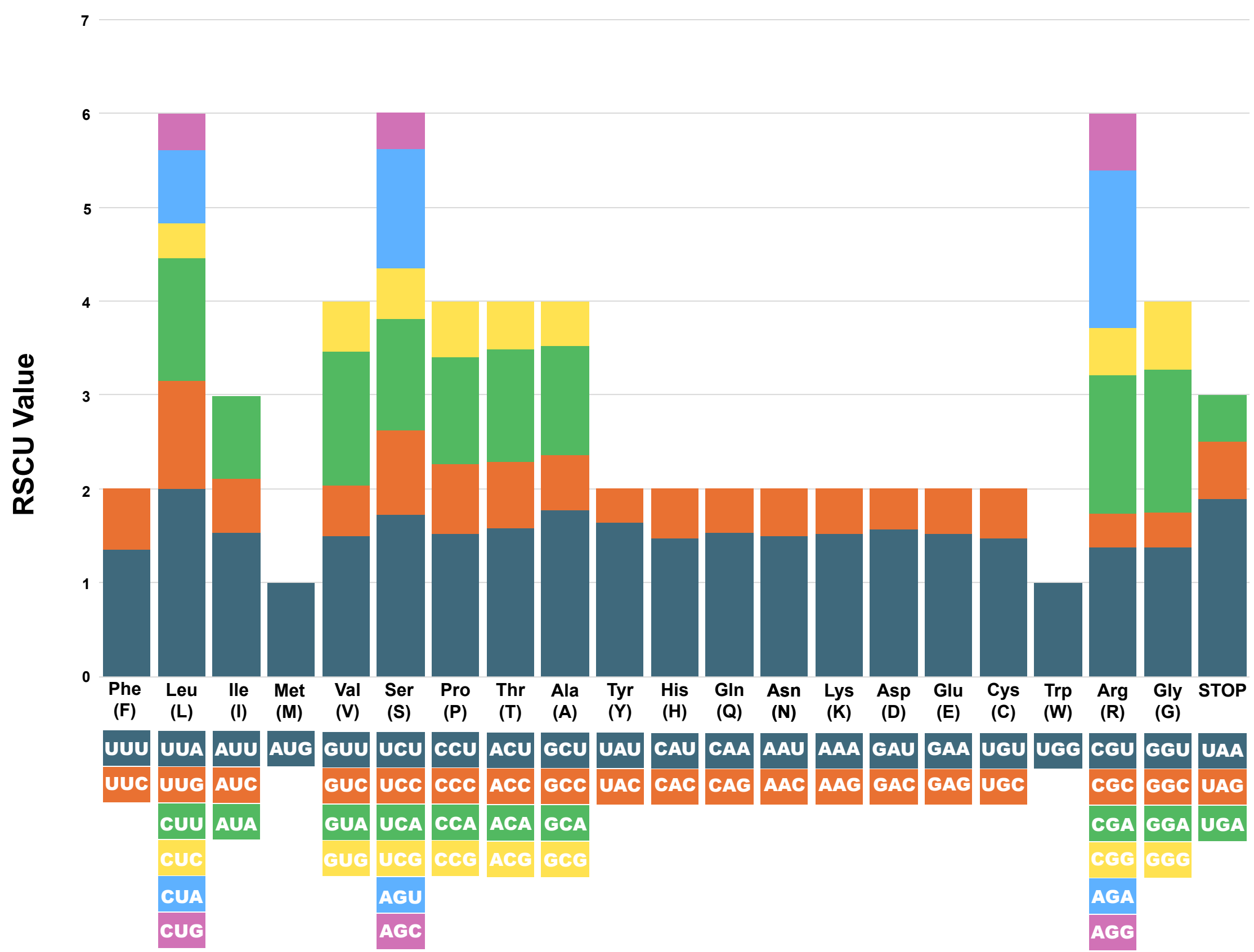

Supplement: Supplementary file 1 [file genes-15-01399-s001.zip › data/figure/Figure_5.pdf]

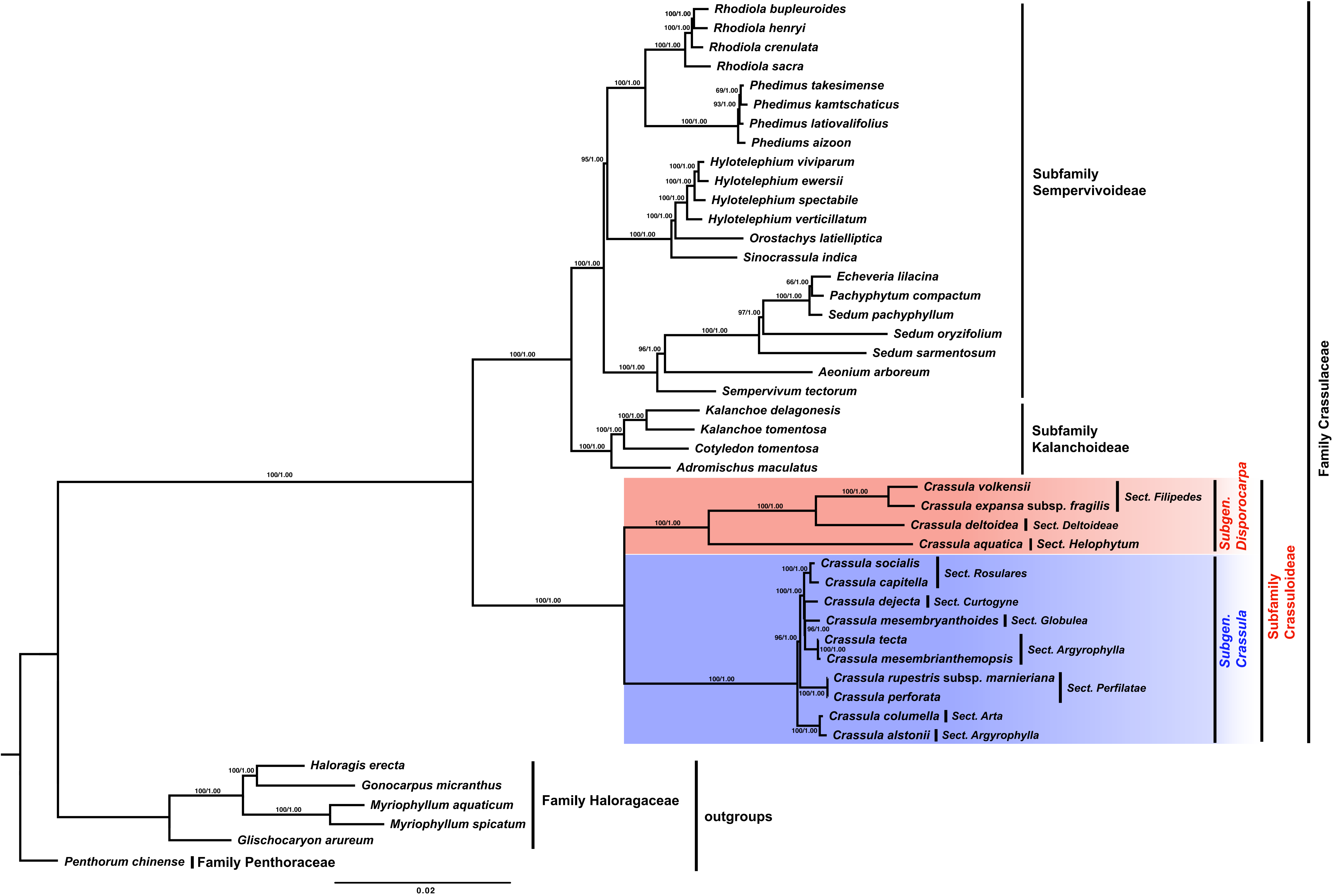

Supplement: Supplementary file 1 [file genes-15-01399-s001.zip › data/figure/FIgure_6.pdf]

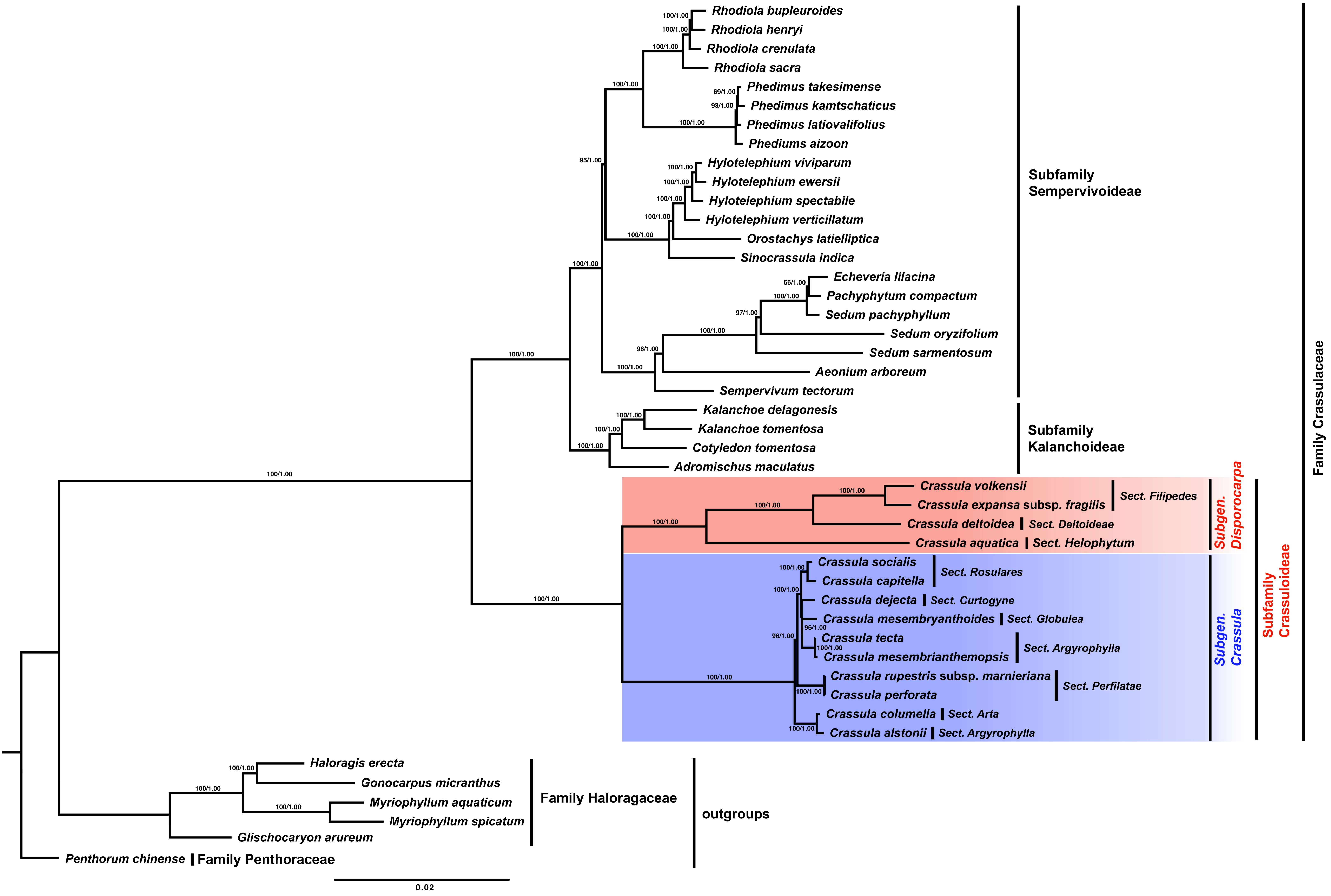

Supplement: Supplementary file 1 [file genes-15-01399-s001.zip › data/figure/FIgure_61.pdf]

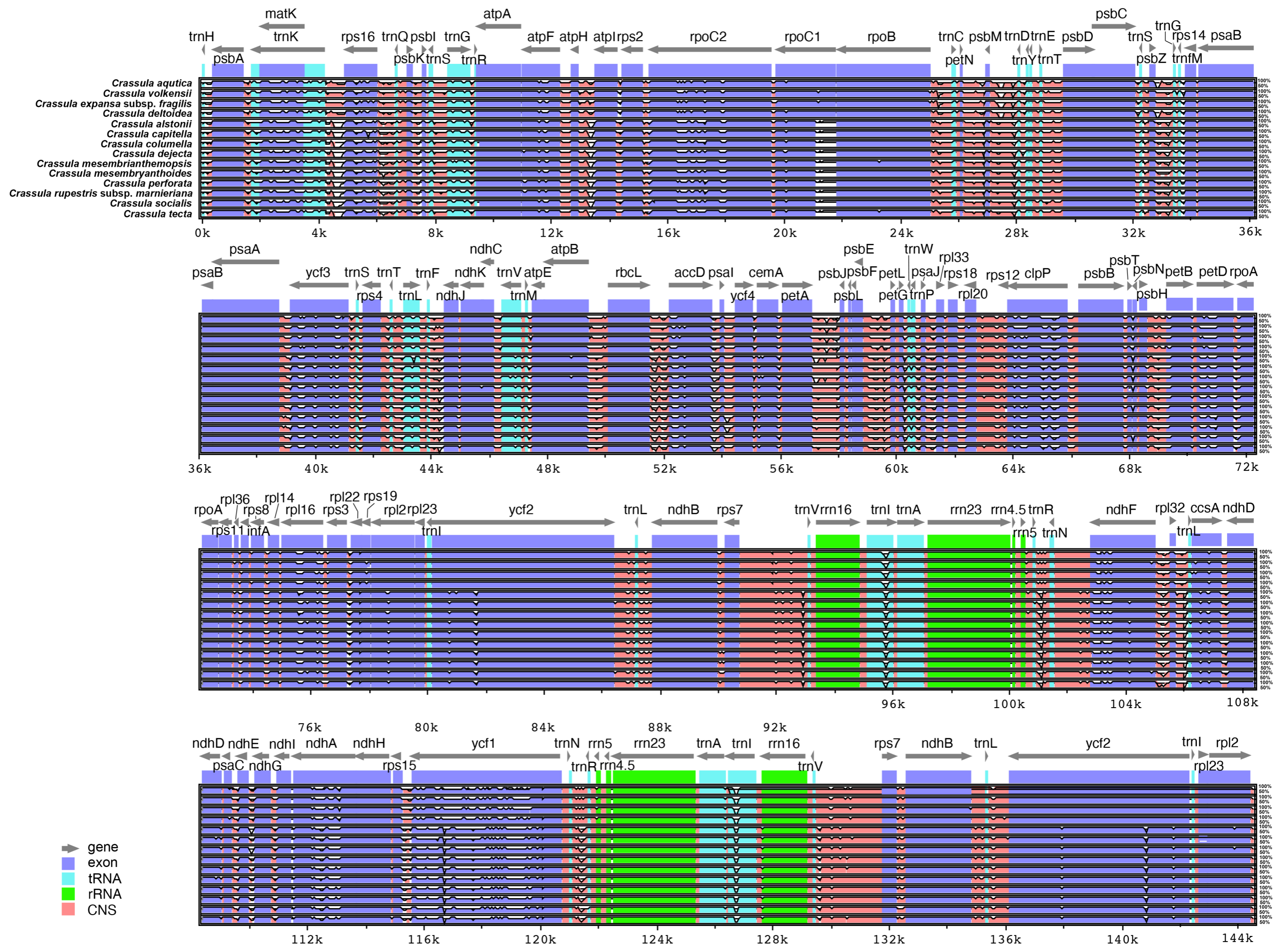

Supplement: Supplementary file 1 [file genes-15-01399-s001.zip › data/figure/Figure_S1.pdf]

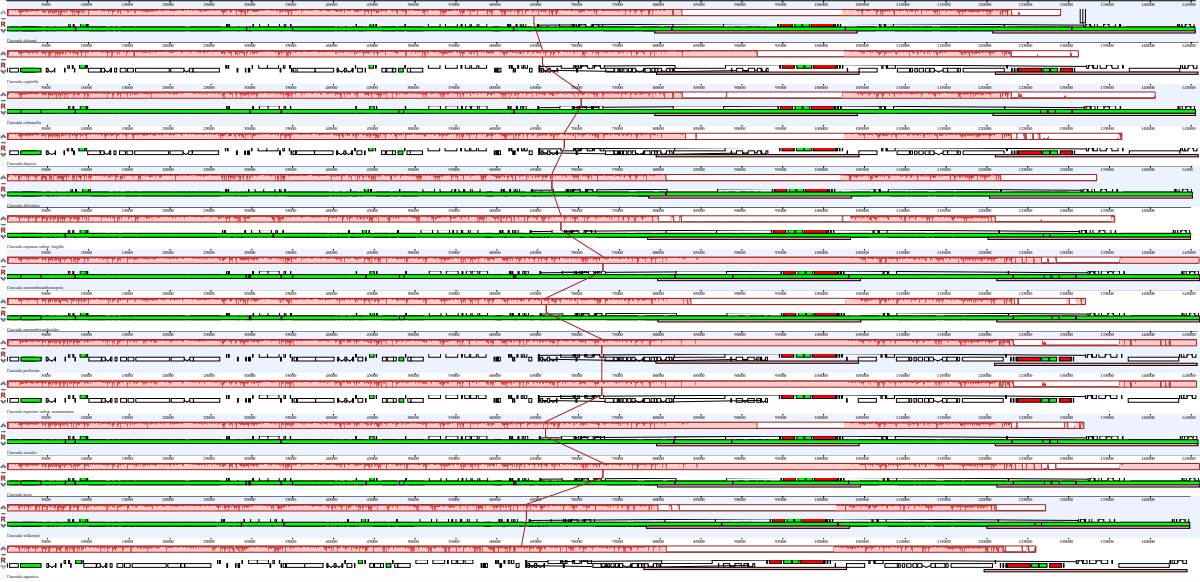

Supplement: Supplementary file 1 [file genes-15-01399-s001.zip › data/figure/Figure_S2.pdf]

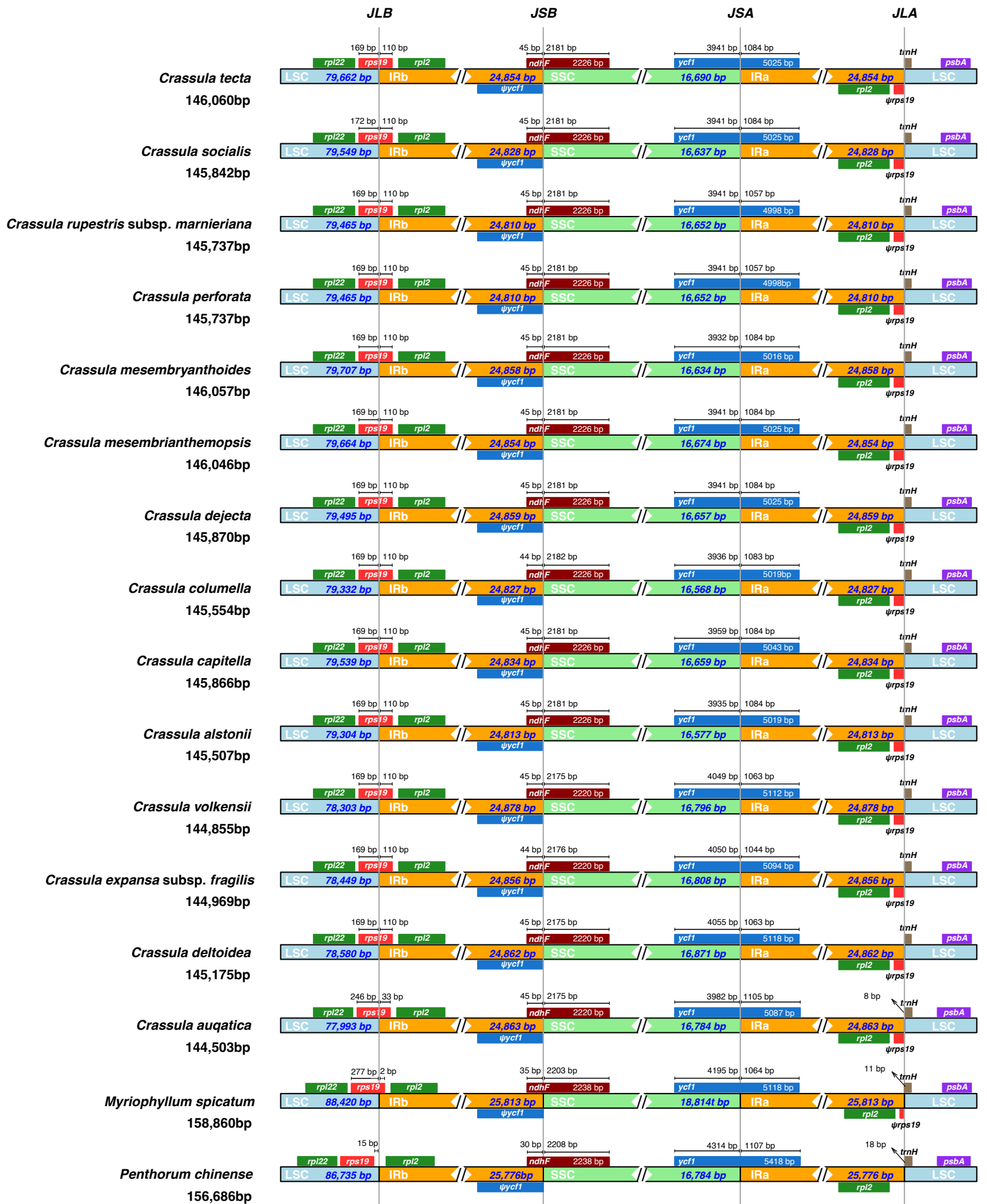

Supplement: Supplementary file 1 [file genes-15-01399-s001.zip › data/figure/Figure_S3.pdf]

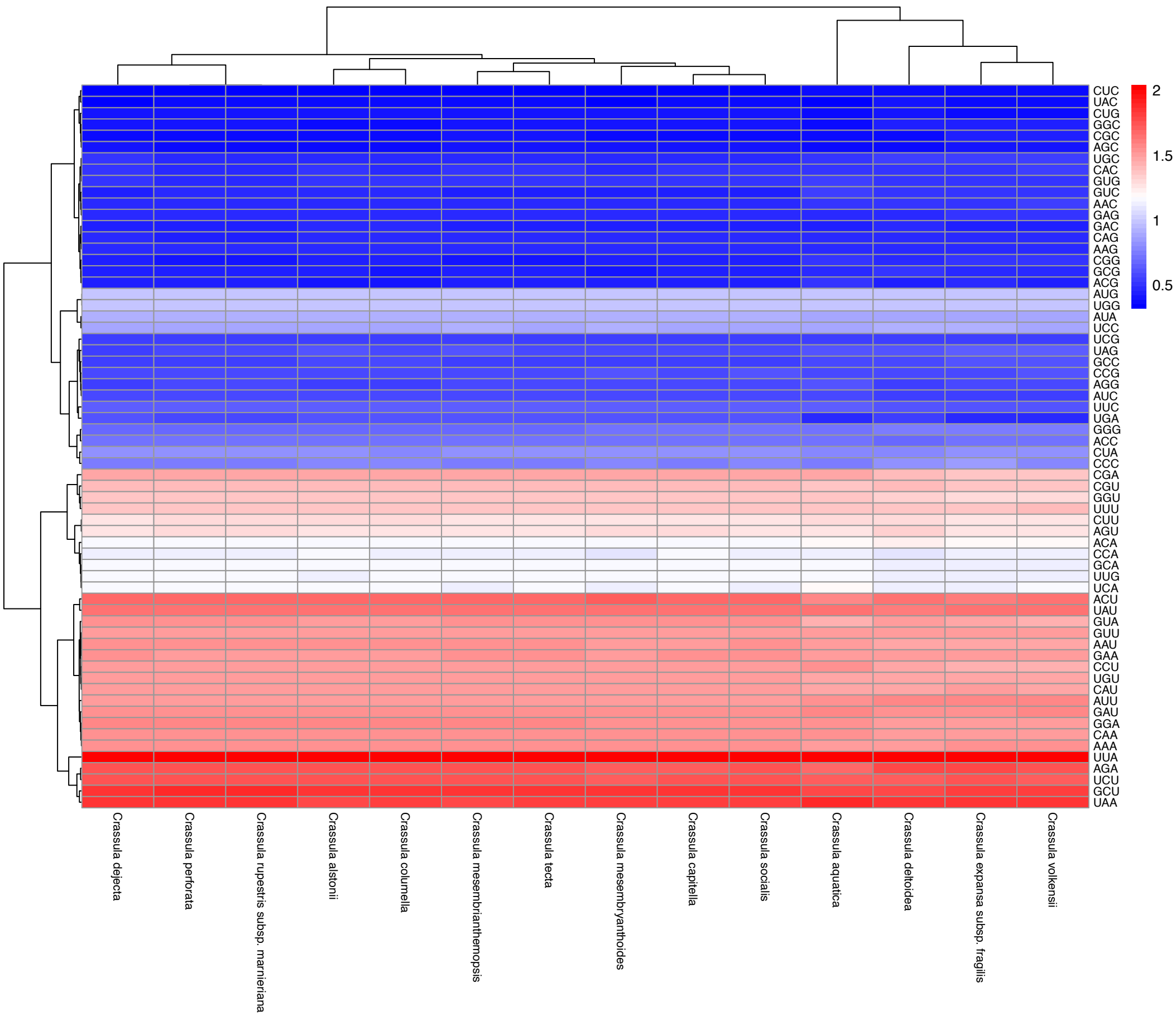

Supplement: Supplementary file 1 [file genes-15-01399-s001.zip › data/figure/Figure_S4.pdf]
